# Unravelling herbicide stress and its impact on metabolite profiling in *Cannabis sativa*: an investigative study

**DOI:** 10.1186/s42238-025-00300-z

**Published:** 2025-07-07

**Authors:** Sabreen Bashir, Navneet Kaur, Agrataben Vadhel, Awadhesh Kumar Verma, Madhuri Girdhar, Tabarak Malik, Anil Kumar, Anand Mohan

**Affiliations:** 1 School of Bioengineering and Biosciences, Lovely Professional University, Phagwara, 144411 Punjab India; 2 Division of Research and Development, Lovely Professional University, Phagwara, 144411 Punjab India; 3 Department of Biomedical Sciences, Institute of Health, Jimma University, Jimma, 00000 Ethiopia; 4 Gene Regulation Laboratory, National Institute of Immunology, New Delhi, 110067 India

**Keywords:** *Cannabis sativa*, Phytocannabinoids, Herbicide stress, DFT, Molecular Docking, THC

## Abstract

**Background:**

*Cannabis sativa L.*, renowned for its versatility in pharmaceutical, textile, and cosmetic industries, is highly susceptible to several agronomic and environmental factors, particularly herbicides. These chemical agents, while commonly used for weed control, can adversely affect plant growth, physiology, and secondary metabolite production. Understanding the plant’s response to such external stressors is essential for optimizing its cultivation and ensuring the quality of its bioactive compounds.

**Methods:**

In our current work, we studied the impact of two herbicides- glyphosate and metribuzin on the morpho-physiological and biochemical characteristics of cannabis plants. The secondary metabolite production analysis was carried out using Gas Chromatography-Mass S pectrometry (GC-MS). Furthermore, in silico studies using molecular modelling and optimization via Density Functional Theory (DFT) were performed, followed by molecular docking.

**Results:**

It was observed that both herbicides greatly impact overall plant productivity including primary and secondary metabolite production. Further, glyphosate treatment caused an increase in fatty acid synthesis while the contrary was observed in case of metribuzin. Also, herbicide stress leads to the synthesis of cannabidivarol and cannabidiol although they were absent in the untreated group. These findings provide crucial insights for optimizing agricultural practices in cannabis cultivation. Moreover, molecular simulation results showed that both metribuzin and glyphosate bind at the active pocket of Tetrahydrocannabinolic acid synthase (THCA synthase) and offer a mechanistic explanation for the observed variations in Δ9 -tetrahydocannabinol (THC) levels by suggesting that both herbicides inhibit THCA synthase activity, contributing to a deeper understanding of herbicide-plant interactions at the molecular level.

**Conclusions:**

Our findings indicate that herbicide stress impacts overall cannabis productivity and alters biosynthesis. The stress notably stimulates the production of cannabidivarol and cannabidiol. In addition, molecular docking studies revealed that metribuzin binds to the same active channel as Cannabigerolic acid (CBGA)- the THC precursor, while glyphosate binds at the entrance, thereby hindering THC production. This multifaceted approach guides sustainable farming strategies and has implications for manipulating cannabinoid profiles in pharmaceutical and other industrial applications.

**Supplementary Information:**

The online version contains supplementary material available at 10.1186/s42238-025-00300-z.

## Introduction

Over the last decade, the prevailing agricultural practice of growing a limited number of crops in monoculture has faced substantial criticism due to its adverse impact on the environment. Both the recent European Union policy and the common worldwide trend emphasize the need for diversified cropping and the revival of numerous abandoned crops. Among such crops, *Cannabis sativa* L. which belongs to the family Cannabaceae, has received heightened attention from farmers (Durak et al. 2021). It is one of the earliest domesticated plants with a cultivation history spanning over 6000 years, substantiated by archeological evidence. It is a multipurpose crop with applications ranging from textile fiber (Réquilé et al. 2021), food (Clarke and Merlin 2016), paper production (Karche and Singh 2019), pharmaceutical products (Legare et al. 2022), cosmetics (de Andrade et al. 2023), biofuel production (Bharath Viswanathan et al. 2020), biomass (energy) (Rehman et al. 2021), and much more(Abdollahi et al. 2020) and can thus be considered a fundamental cash crop.

Cannabis plants contribute significantly to environmental well-being by absorbing up to 10 metric tons of carbon dioxide per vegetation cycle and promoting air quality, thermal balance, and ecological stability. Thus, the agricultural sector is increasingly drawn to cannabis cultivation for its environmental advantages and growing cannabis product market, aligning with sustainable practices and climate change mitigation throughout the entire value chain (Raihan and Bijoy 2023). Cannabis (*Cannabis sativa* L.) is also gaining prominence as an industrial crop due to its large number of applications (Peng and Shahidi 2021). Market liberalization and UN reclassification have driven global expansion, increasing demand for cannabis fiber and derivatives (Guedes et al. 2024; Simiyu et al. 2022). The global cannabis market is projected to grow rapidly, reaching over USD 444 billion by 2030 (Gallien and Occhiali 2023). Cannabis is estimated to possess more than 550 molecules of bioactive compounds (Lowe et al. 2021), belonging to alkaloids, flavonoids, terpenoids, carotenoids, and cannabinoid classes (Andre et al. 2016). Currently, research on *Cannabis sativa* L. is reviving in several disciplines, including microbiology and cancer (Giupponi et al. 2020; Pollastro et al. 2018). These compounds can solve therapeutic issues such as antibiotic resistance and the toxicity that results from ingestion and metabolism while treating cancer (Hourfane et al. 2023). However, various agronomic and environmental factors can influence the cannabis productivity. As the plant’s cultivation expands, it becomes important to understand the effects of commonly applied agrochemicals on its morphological, physiological, and biochemical aspects. 

Today’s agronomy is dependent on herbicide application, which although is greatly advantageous but also poses threats to the biosphere. With the legalization of cannabis cultivation, herbicides are one of the stressors that have the potential to affect its production (Kaur et al. 2025). Among the herbicides which are used globally, glyphosate and metribuzin are widely applied (Kostopoulou et al. 2020). Glyphosate is a broad-spectrum non-specific herbicide with a half-life of 2 to 215 days in soil and 2 to 91 days in water (Singh et al. 2020). Glyphosate inhibits 5-enolpruvylshikimate-3-phosphate synthase (EPSPS) enzyme in the shikimic acid pathway, thereby preventing the synthesis of three aromatic amino acids- phenylalanine, tyrosine, and tryptophan in plants (Liu et al. 2023). These are vital precursors for numerous plant metabolites involved in defense, growth, and stress response (Fuchs et al. 2021). This disruption induces oxidative stress, alters phytohormonal balance, reduces photosynthetic efficiency, and impairs plant-microbe interactions (Eceiza et al.2022a; Gomes et al. 2016). Glyphosate exposure can also lead to lipid peroxidation and degradation of chlorophyll, severely affecting the plant’s biochemical homeostasis (Gomes et al. 2016). On the other hand, metribuzin is a selective triazinone herbicide, used pre- and post-emergence to control broadleaf weeds. It competes with plastoquinone on D1 protein for QB-binding site, therefore blocking the transfer of electrons from QA to QB leading to photooxidation and ultimately causing plant death (Atef Abdel Fatah et al. 2023). Metribuzin is known for its moderate persistence in soil with a 30 to 120-day half-life under field conditions (Maqueda et al. 2009). Its water solubility and leaching properties make it a potential hazard to non-target organisms by contaminating surface and groundwater (Karimmojeni et al. 2022). Metribuzin application has been associated with increased production of reactive oxygen species (ROS), lipid peroxidation, and compromised antioxidant defense systems in several plant species (Kumar et al. 2022). Metabolomic studies further suggest that metribuzin alters amino acid pools and activates salicylate signaling pathways, reflecting substantial physiological shifts under herbicide-induced stress (Kostopoulou et al. 2020).

Increasing evidence supports the notion that environmental and agronomic stress conditions significantly influence cannabinoid biosynthesis in *Cannabis sativa* (Gorelick and Bernstein 2014). Salinity stress has been shown to reduce shoot and bud weight and suppress total THC and CBD yields (Baas and Wijnen2023a). Similarly, nutritional stress such as phosphorus (P) and potassium (K) deficiencies has led to elevated THC concentrations (Song et al. 2023), despite overall lower plant biomass (Saloner and Bernstein2022a; Shiponi and Bernstein 2021a). Conversely, high P levels tend to dilute cannabinoid content, likely due to a yield-dilution effect (Shiponi and Bernstein 2021a). Nitrogen stress also plays a critical role: high ammonium/nitrate (NH₄⁺/NO₃⁻) ratios reduce cannabinoid biosynthesis, whereas low nitrogen conditions were found to increase it (Saloner and Bernstein 2022b). Moreover, abiotic stresses such as drought, UV radiation, and salinity are known to modulate secondary metabolite pathways, providing further insights into the adaptive strategies of cannabis under stress (Gorelick and Bernstein 2017). In addition to environmental stresses, agronomic practices such as planting density (Danziger and Bernstein 2022), and plant shape (Danziger and Bernstein 2021) which affect microclimate conditions in the shoot; and low root salinity levels affected cannabinoid yield (Baas and Wijnen 2023a).

The understanding of cannabis-herbicide interaction is limited, impeding the development of optimized crop protection strategies (Kaur et al. 2025). Glyphosate and metribuzin herbicides are frequently applied during the cannabis season and unintentional exposure through drift is a documented concern for non-target crops (McNaughton et al. 2012). These findings underscore the importance of understanding herbicide-induced stress responses within the broader context of plant-environment interactions. Thus, in the present investigation, these herbicides were sprayed on the groups of cannabis plants chosen for the study to evaluate the potential impact of herbicide drift from surrounding fields on non-target *Cannabis sativa* plants. Further critical analysis was carried out through biochemical studies. Moreover, molecular docking analysis of THCA synthase with glyphosate and metribuzin was performed to validate the results obtained through Gas chromatography-mass spectrometry (GC-MS) analysis for ∆9-Tetrahydrocannabinol (THC) for variation in its synthesis across treatments with different herbicide concentrations. THC is the most powerful psychoactive cannabinoid, and thus, it becomes one of the primary focuses of our investigations (Citti et al. 2019).

## Materials and methods

### Plant material

The *Cannabis sativa* L. seeds were collected from a roadside growing wild plant which was dioecious (female) and initial identification was carried out by Botany Faculty, LPU. Licenses are not required for collection and research working on a wild variety of *Cannabis sativa* in India. The herbarium was prepared and sent to a national referral facility: Janaki Ammal Herbarium CSIR-IIIM. The plant identification was performed by a taxonomist Dr. Sumeet Gairola, at the Council of Scientific and Industrial Research- Indian Institute of Integrative Medicine (CSIR-IIIM) Jammu [accession number = 26 832]. The healthy and uniform seeds were surface sterilized for 15 min using 0.1% Sodium hypochloride. Further, the seeds were rinsed and soaked with distilled water for 20 min. The seeds were germinated uptill 4 leaf stage and plants exhibiting uniform developmental characteristics were transferred in plastic pots containing soil that was not previously treated with any type of chemical. The pots were kept in a greenhouse under controlled conditions at a mean temperature of 24–28℃ in an agricultural field at Lovely Professional University, Phagwara, Punjab. Five weeks later, different concentrations of glyphosate and metribuzin were used to treat the plants. Both glyphosate (active ingredients 40.6% w/w, Agri Venture) and metribuzin (active ingredients 88% w/w, TATA) herbicides used in the current experiment were of industrial grade. The experiment comprised of following treatments: T0 (control), T1 (X/8), T2 (X/4), T3 (X/2), T4(X), where X (2.47 L/ha for glyphosate and 742 g/ha for metribuzin) is the recommended herbicide dose. Each treatment was replicated three times. Glyphosate samples were collected after three days (de Freitas-Silva et al. 2020) of treatment and metribuzin samples after seven days (Kumar et al. 2022) of treatment for morphological analysis and were stored at -20℃ for biochemical analysis.

### Fresh weight, dry weight, and leaf water relations

After harvesting, the fresh weight (FW) of the plants was measured immediately; however, for dry biomass, the plants were placed in an oven set at 80 ± 1.5°C and weighed after five days [30]. Leaf relative water content (RWC) was calculated according to (Barrs and Weatherley 1962), RWC%= (FW − DW)/(TW − DW) x 100, where FW is fresh leaf weight, DW is dry weight and TW is turgid weight after 24 h floating in distilled water at 4 °C in darkness.

### Photosynthetic pigments

Leaf pigments i.e., chlorophyll, carotenoids were extracted with 80% acetone and anthocyanins were extracted with acidified methanol (methanol: water: HCl, 79:20:1). Chlorophyll, carotenoids, and anthocyanins were estimated by methods given by (Arnon 1949), (Maclachlan and Zalik 1963) and (Mancinelli 1984) respectively. The absorbance of supernatant was noted at 645 nm, 663 nm, 480 nm, 510 nm, 530 nm, and 657 nm using UV-vis spectrophotometer (LI-2800 Ex) and calculated through the following formulas: chlorophyll *a* (mg g^− 1^ FW)= (Abs_663_ × 12.7) −(Abs_645_ × 2.69) × V/(1000 × W); chlorophyll *b* (mg g^− 1^ FW)= (Abs_645_ × 22.9) − (Abs_663_ × 4.68) × V/(1000 × W); total chlorophyll content (mg g^− 1^ FW)= (Abs_645_ × 22.2) + (Abs_663_ × 8.03) × V/(1000 × W); total carotenoid content (mg g^− 1^ FW) = 1000 (Abs_480_) − 1.8 (Chl *a*)– 85.02 (Chl *b*)/ 198; anthocyanin content = Abs_530_– 0.25(Abs_657_).

### Protein and sugar Estimation

Protein content was determined using supernatant obtained through the method by (Lowry et al. 1951) by plotting its absorbance against the BSA standard. Subsequently, Total Soluble Carbohydrate was estimated according to (Islam et al. 2021) and sucrose content was determined according to (van Handel 1968). 25 mg of leaf sample was homogenized in 95% of 5 mL ethanol, followed by centrifugation (3500 rpm), and the supernatant was used for sugar estimation. Glucose and sucrose were used to plot the standard curve by taking absorbance at 625 nm and 620 nm.

### Antioxidant enzyme activity

In a pre-chilled pestle and mortar, 0.1 g of plant material was crushed using 3 mL of 100 mM potassium phosphate buffer (PPB) at a pH of 7.0. Then the crushed material was centrifuged for 20 min at 13,000 rpm and 4°C. The supernatant was then collected to calculate various antioxidant activities. Peroxidase (POD) activity, catalase activity (CAT), and Malondialdehyde (MDA) contents were determined by the methods given by (Pütter 1974), (Aebi et al. 1983), and (Heath and Packer 1968) respectively.

### Secondary metabolite Estimation

The conventional method of maceration extraction was utilized to examine the bioactive compounds found in cannabis leaves (Pande and Chanda 2020). Fresh cannabis leaves were air-dried at room temperature (below 24°C) in a shaded and ventilated area before being ground into fine powder for extraction. 1 g of dried leaf powder was then extracted at room temperature for 24 h in 100 mL of methanol by placing it in shaking incubator. The mixture was then paper-filtered and kept at room temperature until the excess solvent evaporated. After getting the extract, it was kept at 4°C in an airtight container for secondary metabolite estimation. The extract was then reconstituted in methanol and filtered through 0.22 micron nylon syringe filter (Moxcare labware) before injecting into the GC. The analysis was conducted at Lovely Professional University’s Central Instrumentation Facility using a Shimadzu gas chromatograph instrument model, GCMS-TQ8040 NX, equipped with a mass detector. 1µL of sample was injected into GC via autosampler. The injector was used in splitless mode. Additionally, the injector was set at a temperature of 250℃. The compounds were separated using an SH-RXi-5Sil MS capillary column and crosslinked to a 30 m × 0.25 mmID, 0.25 μm df (similar to 5% diphenyl/95%dimethyl polysiloxane). The column temperature was initially set at 40°C and then it was raised to 300°C at a rate of 7°C per minute and maintained for three minutes. The carrier gas utilized was helium (flow rate = 1 mL min^− 1^). The National Institute of Standards and Technology’s spectrum database was used to find and identify the components in the extract. Within the m/z range of 40 to 1000, detection was carried out using the Q3 scan acquisition mode. The proportion of an individual component is presented as a relative peak area percentage of the total peak percentage (Vadhel et al. 2023).

### Molecular Docking studies

#### Protein Preparation

The protein 3D structure of the *Cannabis sativa* L. tetrahydrocannabinolic acid synthase (THCA synthase) was acquired from the Protein Data Bank (PDB ID 3VTE), with a resolution of 2.75 Å having no mutation (Shoyama et al. 2012). The flavine adenine dinucleotide (FAD) ligand was kept as it is responsible for the enzyme’s catalytic activity, and the protein was cleaned by removing other ligands and water molecules using AutoDockTools 1.5.6. Polar hydrogens and Kollmann charges were added, and Gasteiger charges were computed. The atomic type was set to assign AD4 type (Verma et al. 2023a).

#### Ligand Preparation

The 3D canonical structures of ligands- Cannabigerolic acid (CBGA), metribuzin, and glyphosate, were drawn using Marvin sketch software. 2D and 3D cleaning was performed each time, and structures were checked for 3D geometrical conformation by visualizing them in Marvin’s view. Further, the structures were optimized to transition state, and the energy was minimized sequentially using Chem3D Pro 12.0.2, Avogadro 1.2, and Chem3D 22. The ligand with the most minimized energy was finally optimized using DFT via Gaussian 09 software. Here, we utilized RB3LYP functionals and a 6-311G basis set. These optimized structures were further used for docking purposes to determine the interactions of ligands with the enzyme THCA Synthase (Gulati et al. 2023).

#### Molecular Docking

The Rigid molecular docking experiment was performed with optimized ligands using Autodock 4.2. Grid box dimensions were set to cover the whole protein at a distance of 0.714 Å (Supplementary Table S1). The Genetic Algorithm simulation program was used with a 300 population size, 100 genetic algorithm runs, and a medium number of evaluations for metribuzin and glyphosate due to a medium number of torsions, whereas a long number of evaluations were used for Cannabigerolic acid (CBGA) due to a higher number of torsions in CBGA, and the remaining parameters were set to their default values. The output file was saved as Lamarckian GA. Each ligand’s top 10 conformations were saved based on the negative binding energy (∆G). Conformations binding to the same active site and the most negative binding energy in all three were further analyzed in Discovery Studio Visualizer v21.1 and ChimeraX 1.7 (Verma et al. 2022, 2023a).

### Statistical analysis

The collected data was analyzed using one-way Analysis of Variance (ANOVA) with a significance level of ≤ 0.05. Duncan multiple comparison test was conducted using IBM SPSS version 22 software. The different letters on each error bar are statistically significant. Graphs were plotted using OriginLab software. The chemical structures were drawn with ChemAxon Marvin Sketch software.

## Result

### Fresh weight, dry weight, and relative water content

**Fig. 1:**
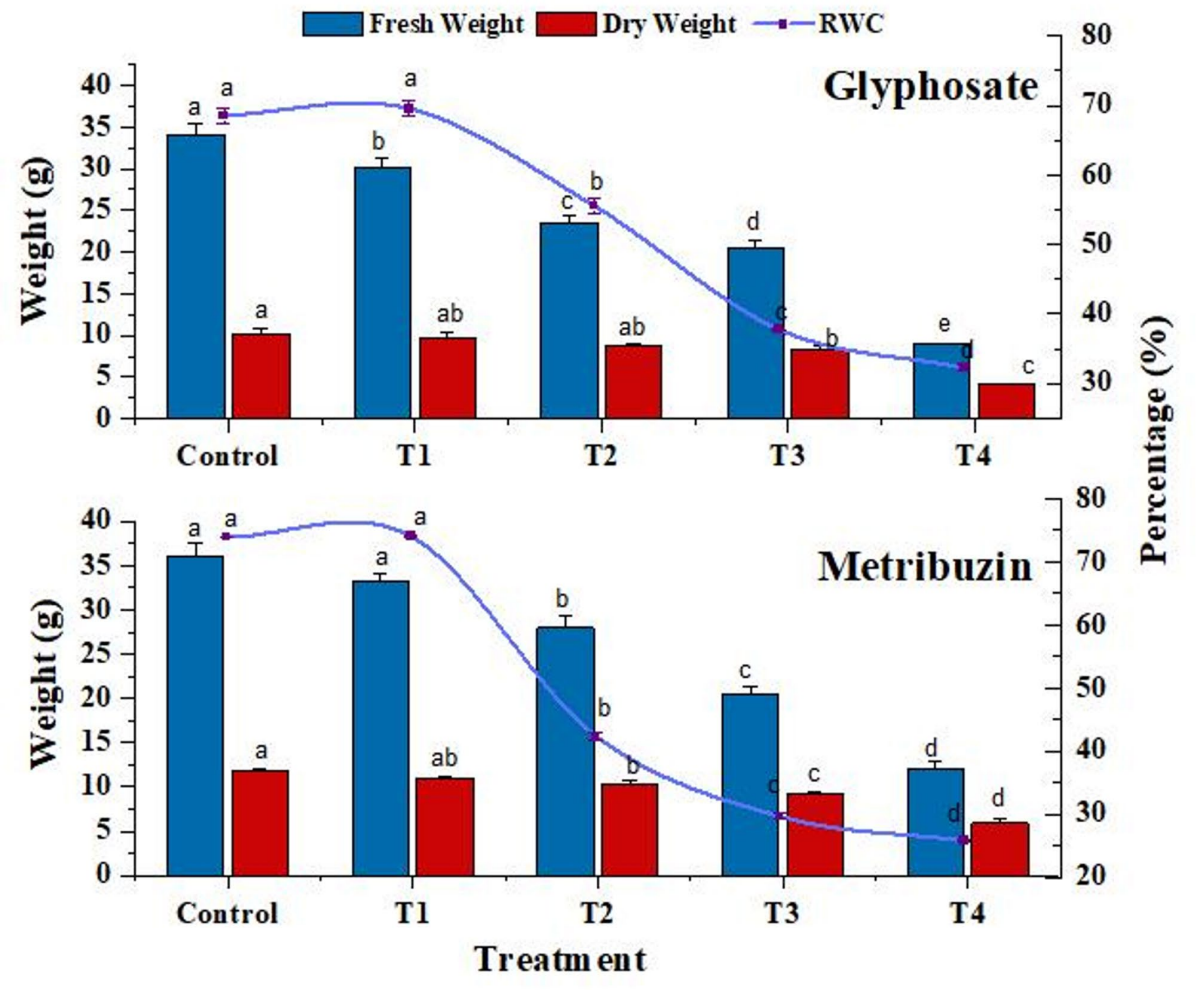
Change in fresh weight (g), dry weight (g), and Relative Water Content (RWC) (%) in control and treated plants. Data represent mean values (± standard error) of three replicates (*n* = 3). Treatments are as follows: T1 = X/8, T2 = X/4, T3 = X/2, and T4 = X, where X is 2.47 L/ha for glyphosate and 742 g/ha for metribuzin. Different letters above bars indicate statistically significant differences among treatments for each pigment, as determined by one-way ANOVA followed by Duncan’s multiple range test (*p* ≤ 0.05)

Increased herbicide stress led to a significant reduction in fresh and dry weights, with glyphosate and metribuzin exposure compared to the control at the highest concentration (T4). The RWC of leaves markedly decreased in both herbicide treatments, reaching its lowest value at T4 (30.3% for glyphosate and 28.6% for metribuzin) (Fig. 1).

### Photosynthetic pigments

**Fig. 2:**
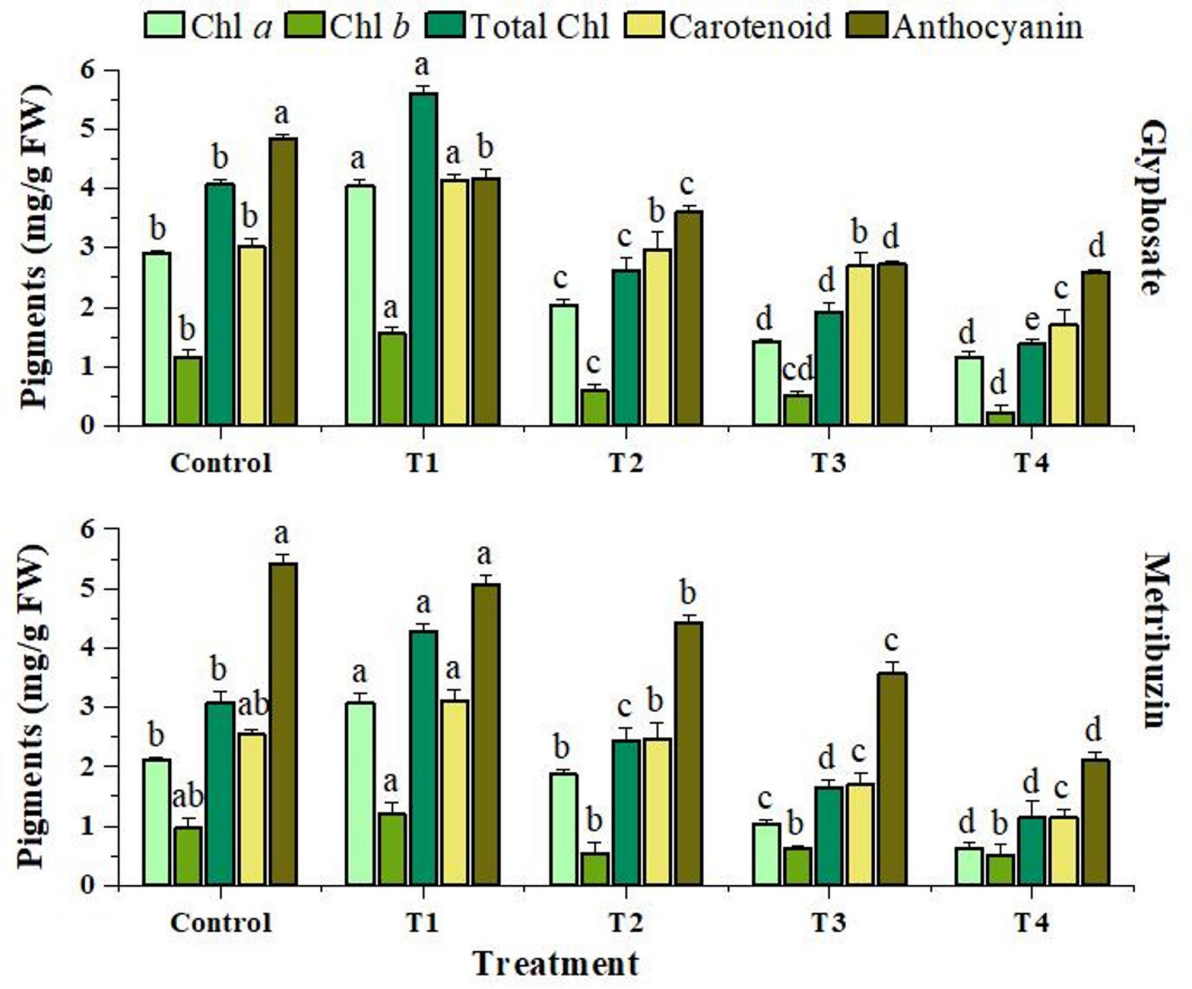
Effect of glyphosate and metribuzin on photosynthetic pigments of *Cannabis sativa* L. Data represent mean values (± standard error) of three replicates (*n* = 3). Treatments are as follows: T1 = X/8, T2 = X/4, T3 = X/2, and T4 = X, where X is 2.47 L/ha for glyphosate and 742 g/ha for metribuzin. Different letters above bars indicate statistically significant differences among treatments for each pigment, as determined by one-way ANOVA followed by Duncan’s multiple range test (*p* ≤ 0.05)

The photosynthetic pigments showed a significant fold reduction in levels of Chlorophyll *a* (2.51, 3.34), Chlorophyll *b* (5.2, 1.89), total Chlorophyll (2.95, 2.69), and carotenoids (1.77, 1.64) under highest glyphosate and metribuzin herbicide stress (T4) as compared to the control group. However, at the lowest concentration (T1), a notable increase was observed in the levels of pigments. Anthocyanin content also showed a decline with increasing stress of 1.8-fold in glyphosate and 1.12-fold in metribuzin at T4 (Fig. 2).

### Protein content

**Fig. 3:**
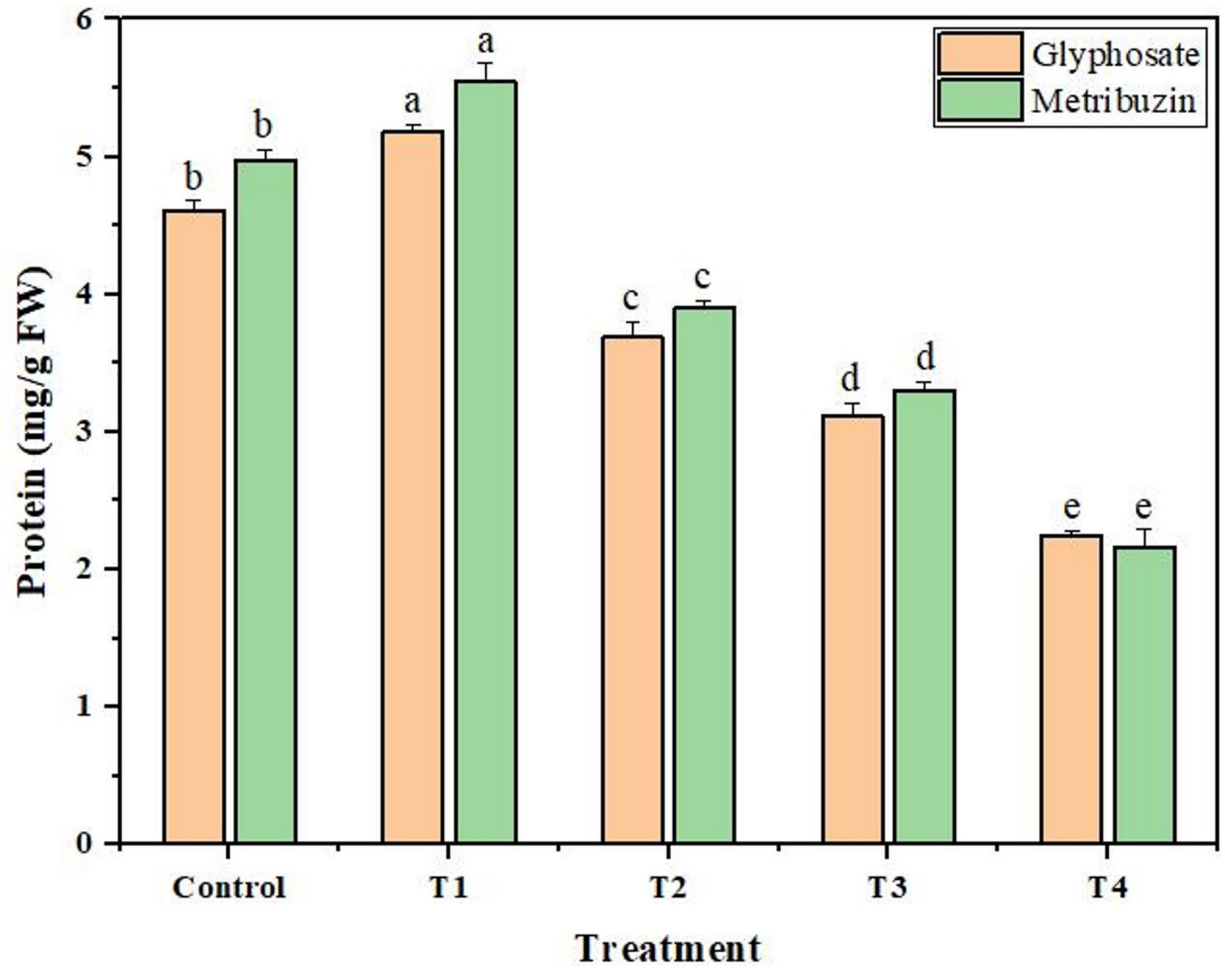
Change in protein content of *Cannabis sativa* L. after glyphosate and metribuzin treatment. Data represent mean values (± standard error) of three replicates (*n* = 3). Treatments are as follows: T1 = X/8, T2 = X/4, T3 = X/2, and T4 = X, where X is 2.47 L/ha for glyphosate and 742 g/ha for metribuzin. Different letters above bars indicate statistically significant differences among treatments for each pigment, as determined by one-way ANOVA followed by Duncan’s multiple range test (*p* ≤ 0.05)

The protein content declined in correlation with an increase in concentrations of both glyphosate and metribuzin. The maximum reduction in protein content reaches 2.04-fold at T4 − the highest glyphosate stress level. Similarly, with the increase in metribuzin concentration, the protein content was observed to decrease, culminating in a maximum reduction of 2.29-fold at the most intensive metribuzin stress point − T4. This indicates a consistent pattern of decreasing protein content with heightened herbicide concentrations except at the lowest doses where a 1.12-fold and 1.11-fold increase in protein content was observed for glyphosate and metribuzin respectively in comparison to the untreated group (Fig. 3).

### Antioxidant enzymes

**Fig. 4:**
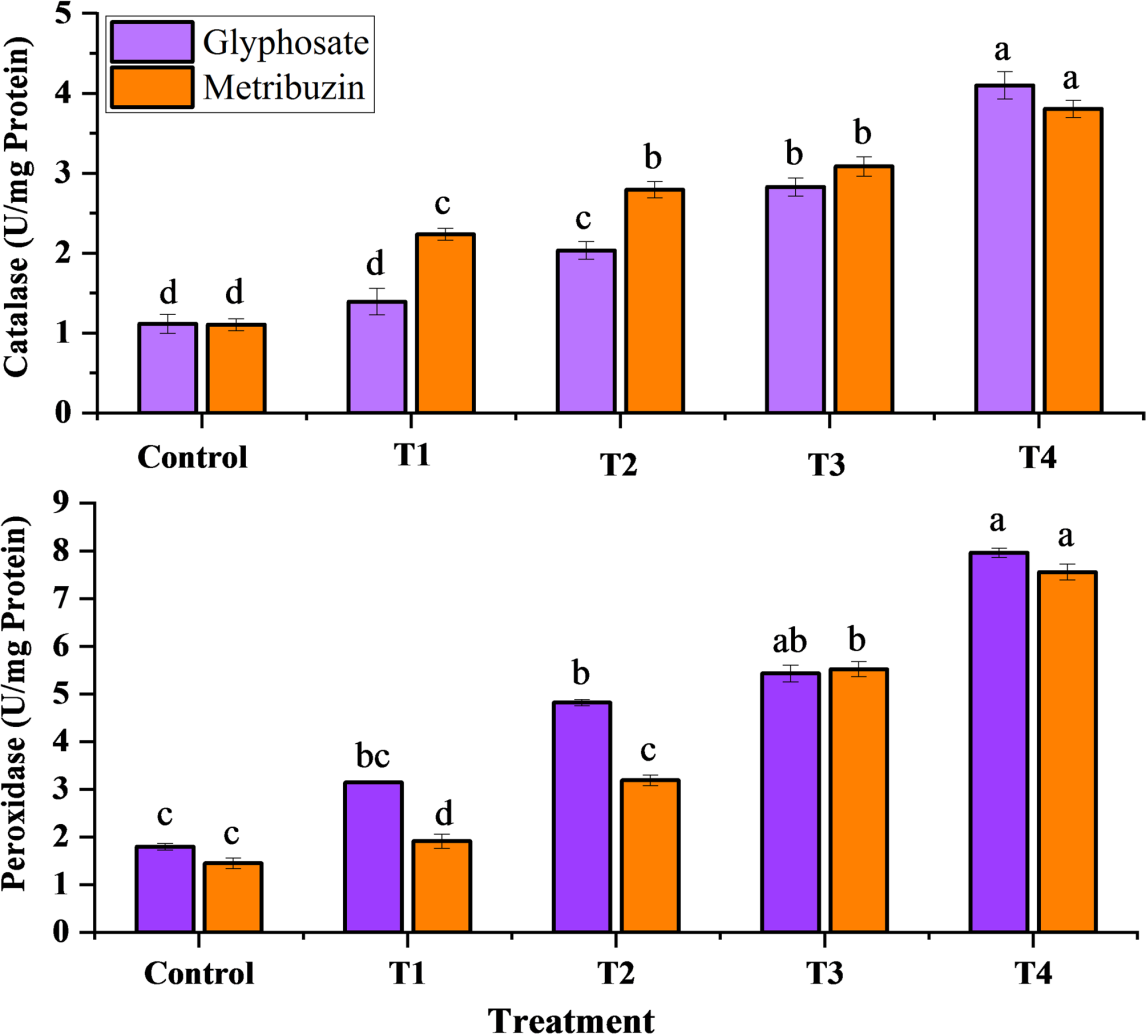
Variation in enzyme activity of *Cannabis sativa* L. under glyphosate and metribuzin herbicide stress. Data represent mean values (± standard error) of three replicates (*n* = 3). Treatments are as follows: T1 = X/8, T2 = X/4, T3 = X/2, and T4 = X, where X is 2.47 L/ha for glyphosate and 742 g/ha for metribuzin. Different letters above bars indicate statistically significant differences among treatments for each pigment, as determined by one-way ANOVA followed by Duncan’s multiple range test (*p* ≤ 0.05)

**Fig. 5:**
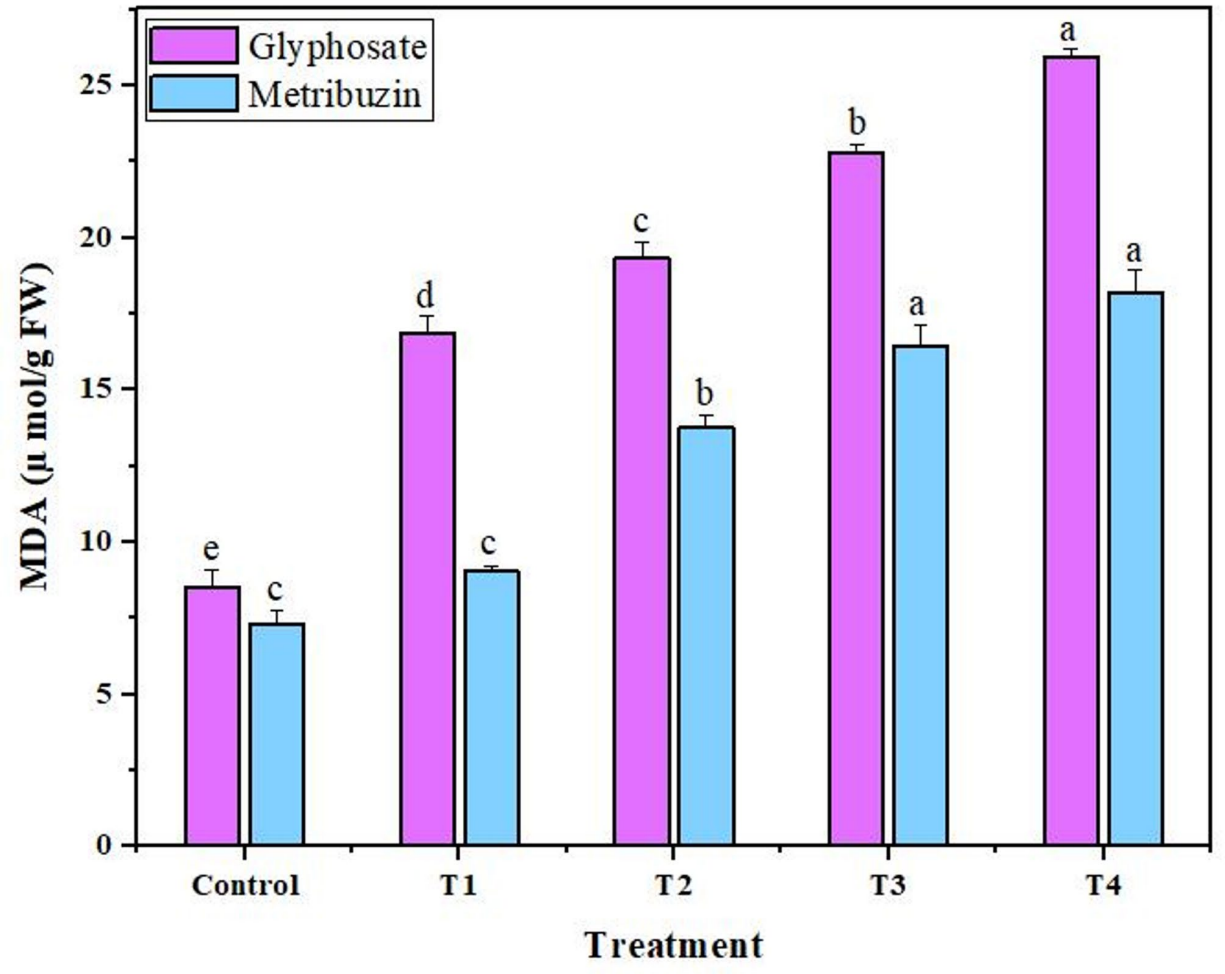
Increase in MDA after application of glyphosate and metribuzin. Data represent mean values (± standard error) of three replicates (*n* = 3). Treatments are as follows: T1 = X/8, T2 = X/4, T3 = X/2, and T4 = X, where X is 2.47 L/ha for glyphosate and 742 g/ha for metribuzin. Different letters above bars indicate statistically significant differences among treatments for each pigment, as determined by one-way ANOVA followed by Duncan’s multiple range test (*p* ≤ 0.05)

The observed data indicated a dose-dependent escalation in peroxidase and catalase activities for both glyphosate and metribuzin treatments. The most substantial enhancement in catalase and peroxidase activities, reaching 3.6-fold and 3.7-fold for glyphosate, and 3.4-fold and 5.2-fold for metribuzin compared to the control, was notably evident at T4, signifying the highest response to oxidative stress (Fig. 4). Simultaneously, the Malondialdehyde (MDA) levels, serving as an index of oxidative damage, consistently rose with increasing concentrations of the herbicides. At T4, MDA also exhibited its highest content, displaying an increase of 3.0-fold and 2.4-fold compared to the control, underlining the culmination of oxidative stress at the highest concentration levels of both glyphosate and metribuzin respectively (Fig. 5).

### Carbohydrate and sucrose

**Fig. 6:**
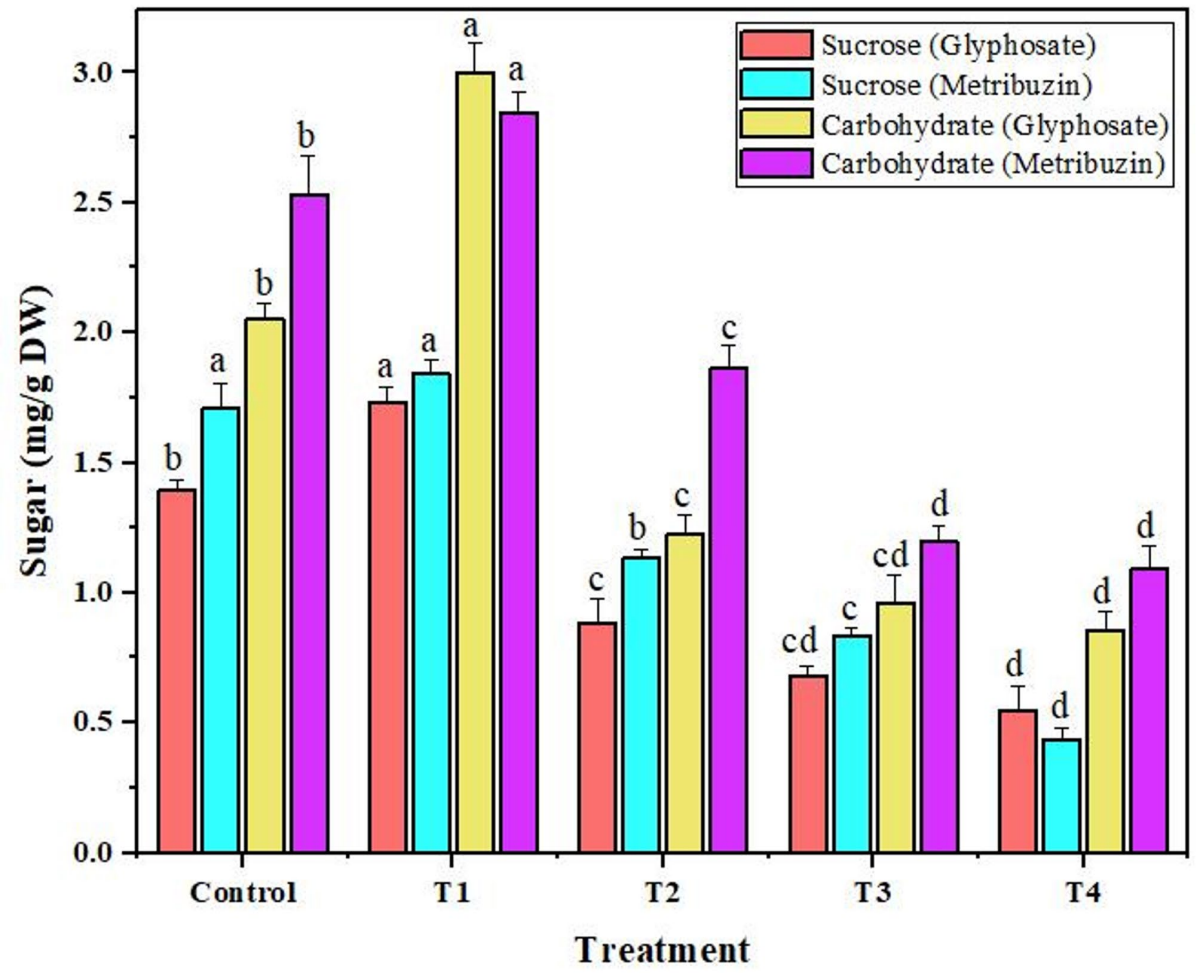
Variation in sucrose and carbohydrate in *Cannabis sativa* L. under glyphosate and metribuzin herbicide stress. Data represent mean values (± standard error) of three replicates (*n* = 3). Treatments are as follows: T1 = X/8, T2 = X/4, T3 = X/2, and T4 = X, where X is 2.47 L/ha for glyphosate and 742 g/ha for metribuzin. Different letters above bars indicate statistically significant differences among treatments for each pigment, as determined by one-way ANOVA followed by Duncan’s multiple range test (*p* ≤ 0.05)

In accordance with the behavior of photosynthetic pigments, the levels of carbohydrate and sucrose mirrored a similar trend when subjected to varying herbicide concentrations. Both compounds displayed a consistent decrease as the herbicide concentration increased, aligning with the anticipated impact on these plant sugars. An interesting deviation from this trend emerged at the lowest concentration (T1), where a slight increase of 1.4-fold and 1.2-fold (glyphosate) and 1.12-fold and 1.07-fold (metribuzin) was observed in both carbohydrate and sucrose levels respectively. Remarkably, when exposed to glyphosate, the most significant reduction percentages were noted at the T4, measuring 2.3-fold for carbohydrates and 2.5-fold for sucrose. Comparatively, with metribuzin application, the reduction percentages were recorded at 2.3-fold for carbohydrates and 3.9-fold for sucrose at the same T4 concentration (Fig. 6).

### Bioactive compound identification

The methanolic leaf extract of the cannabis plant comprised various compounds identified (Supplentary Table S6). Among the identified compounds, the most abundant compounds were hexadecenoic acid, methyl stearate, 2,4-Di-tert-butylphenol (2,4-DTBP), and eicosane. The most important phytocannabinoids included delta.9-tetrahydrocannabivarin (THCV), dronabinol (THC), cannabinol (CBN), Cannabidivarol (CBDV), and cannabidiol (CBD). Dronabinol is the synthetic active enantiomer of Δ9 -tetrahydocannabinol (THC) which is a plant-derived cannabinoid (Devinsky et al. 2015). Therefore, in this paper, the word THC has been used in place of dronabinol. Other compounds including heptadecane, hexadecane, heneicosane, docosane, and tetracosane were also found in the extract in trace amounts (Supplementary Figure S1).

### Semi-quantitative analysis of bioactive compounds from C. *sativa* L

**Fig. 7:**
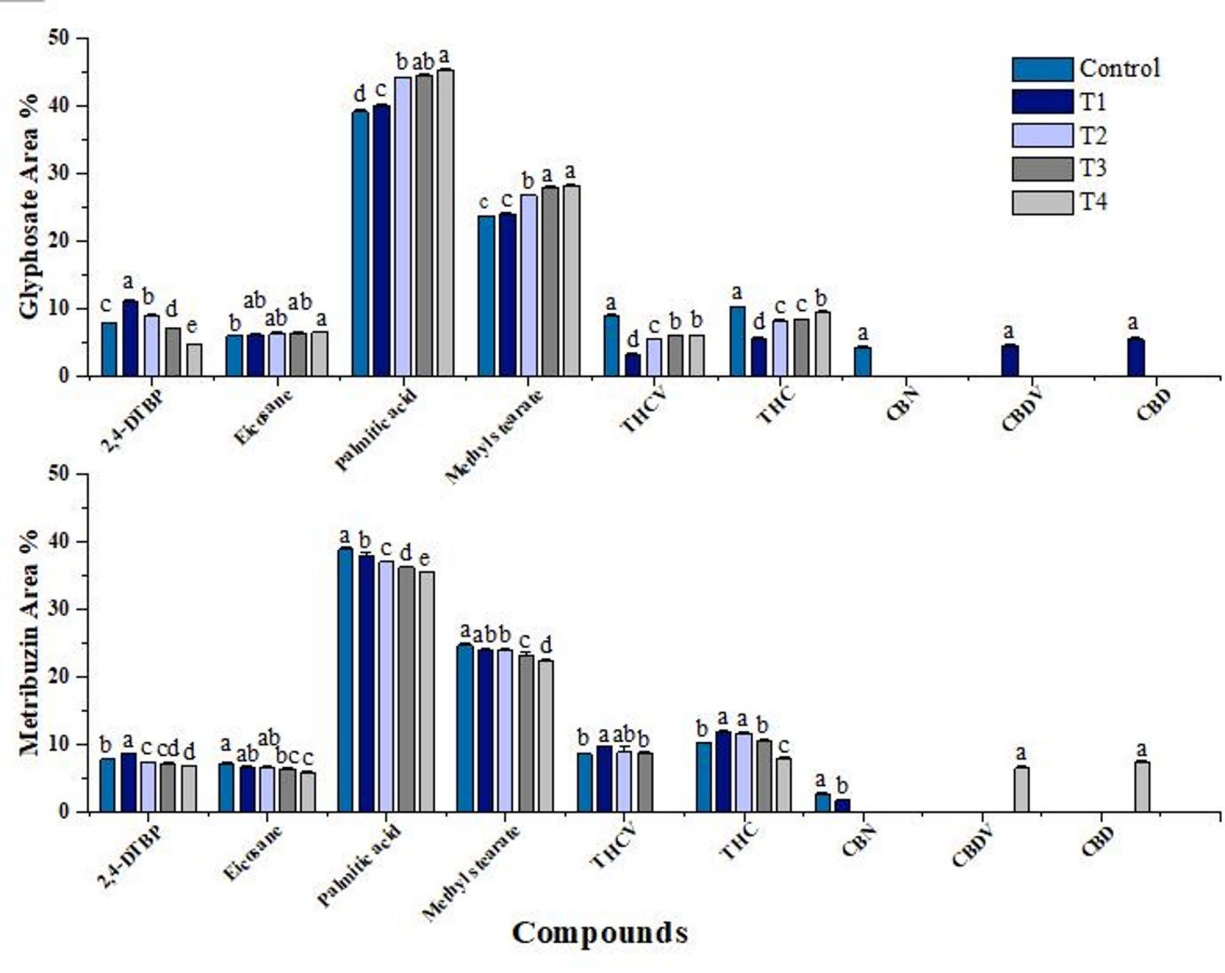
Percent relative peak area value of compounds found in *Cannabis sativa* L. under different glyphosate and metribuzin herbicide stress concentrations. Data represent mean values (± standard error) of three replicates (*n* = 3). Treatments are as follows: T1 = X/8, T2 = X/4, T3 = X/2, and T4 = X, where X is 2.47 L/ha for glyphosate and 742 g/ha for metribuzin. Different letters above bars indicate statistically significant differences among treatments for each pigment, as determined by one-way ANOVA followed by Duncan’s multiple range test (*p* ≤ 0.05)

2,4-Di-tert-butylphenol (2,4-DTBP), an allelopathic component identified in cannabis, has shown significant variation when exposed to different concentrations of glyphosate and metribuzin herbicides. Interestingly, an increase in area percent of 2,4-DTBP was noted at lower concentrations of both herbicides, which gradually decreased as the herbicide stress increased, indicating a direct link between herbicide stress and 2, 4-DTBP biosynthesis (Fig. 7).

A dynamic profile of fatty acid methyl esters, including methyl stearate, hexadecanoic acid, hexadecane, heptadecane, eicosane, etc. was also observed in GC-MS analysis of leaf extract of cannabis. The glyphosate-induced stress led to increase in fatty acids area percentage (Fig. 7). Consequently, stress induced by metribuzin had an inverse effect, resulting in a decline of the fatty acids.

The presence of cannabinoids including THCV, THC, cannabinol, Cannabidivarol, cannabidiol, etc. were also observed. The present data revealed an increase in area percent of THCV and THC under glyphosate stress while metribuzin stress exhibited a negative correlation (Fig. 7). However, the results of THC show some inconsistencies as in the case of metribuzin treatment, the THC area percent in T1 is higher which can be explained by hormesis. On the other hand, glyphosate resulted in a decrease in THC at the lowest concentration. The cannabinol content was observed exclusively in the control group and the lowest concentration of metribuzin. In contrast, Cannabidivarol and cannabidiol were absent in the control group but were present at the lowest concentration of glyphosate and the highest concentration of metribuzin.

### Ligand structural comparison and optimization

This study conducted a comparative analysis of the physicochemical characteristics of CBGA, metribuzin, and glyphosate (Supplementary Table S2). The aromatic ring (AR) is responsible for π − π interactions with amino acid residues, which are present in both CBGA and metribuzin leading to an increase in binding affinity and influencing biological activity. Glyphosate has the highest hydrogen bond acceptors (HBA) and hydrogen bond donors (HBD) which positively influence the number of hydrogen bonds between the ligand and the enzyme THCA Synthase; however, the number of rotatable bonds (RB) in metribuzin is lower than in glyphosate. Furthermore, metribuzin has a slightly greater molecular weight (MW) compared to glyphosate.

After initial energy minimization using several software programs, CBGA, and metribuzin still exhibited positive ∆G energy, while glyphosate demonstrated negative ∆G energy (Supplementary Table S3). Upon Gaussian optimization, all three ligands exhibited minimal negative ∆G energy (Supplementary Figure S2, S3, S4). CBGA had the lowest energy, followed by metribuzin and glyphosate (Supplementary Table S4). Upon optimization, the bond length and angle of CBGA and metribuzin exhibited minor modifications; however, in the case of glyphosate, considerable alterations were observed (Supplementary Table S5).

### Molecular Docking

**Fig. 8:**
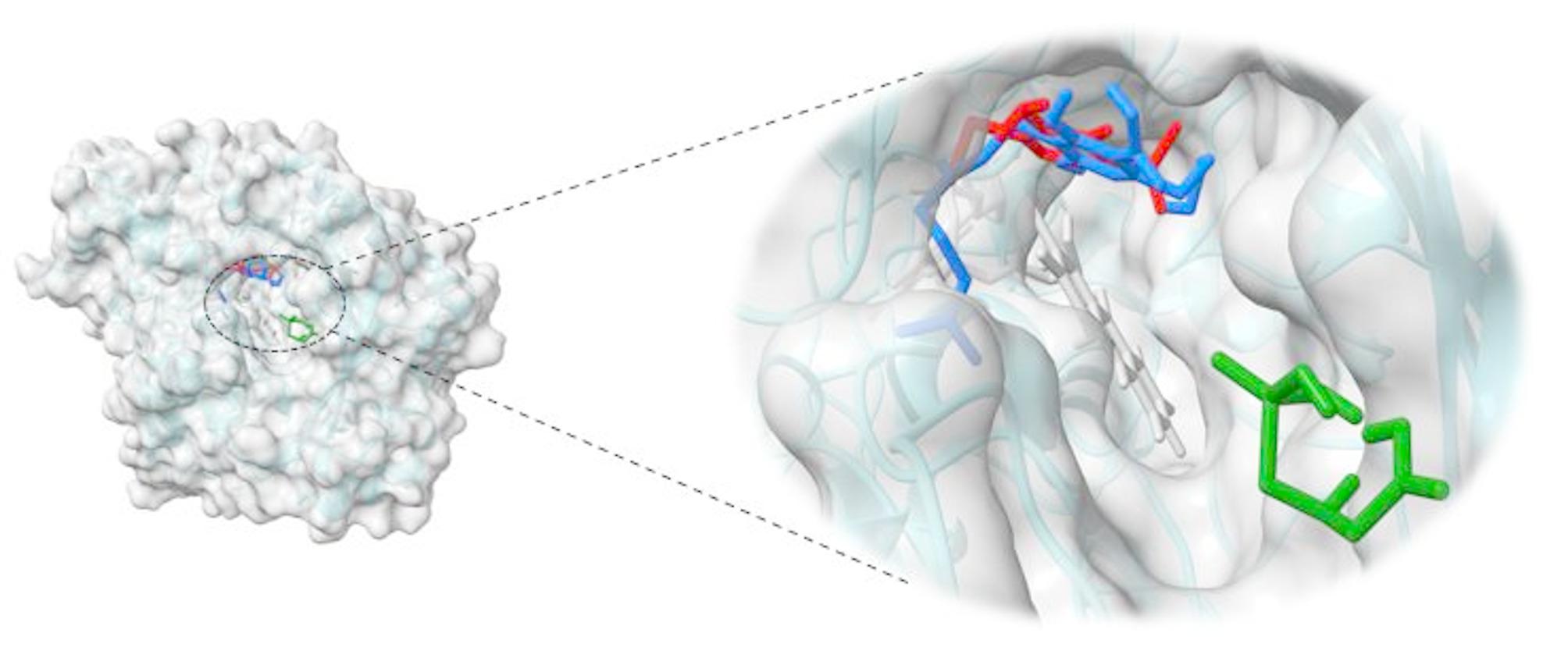
Three-dimensional surface view of THCA synthase and enlarged view of docked ligands CBGA (blue), Metribuzin (red), and Glyphosate (green) at the active channel containing FAD (grey)

**Fig. 9:**
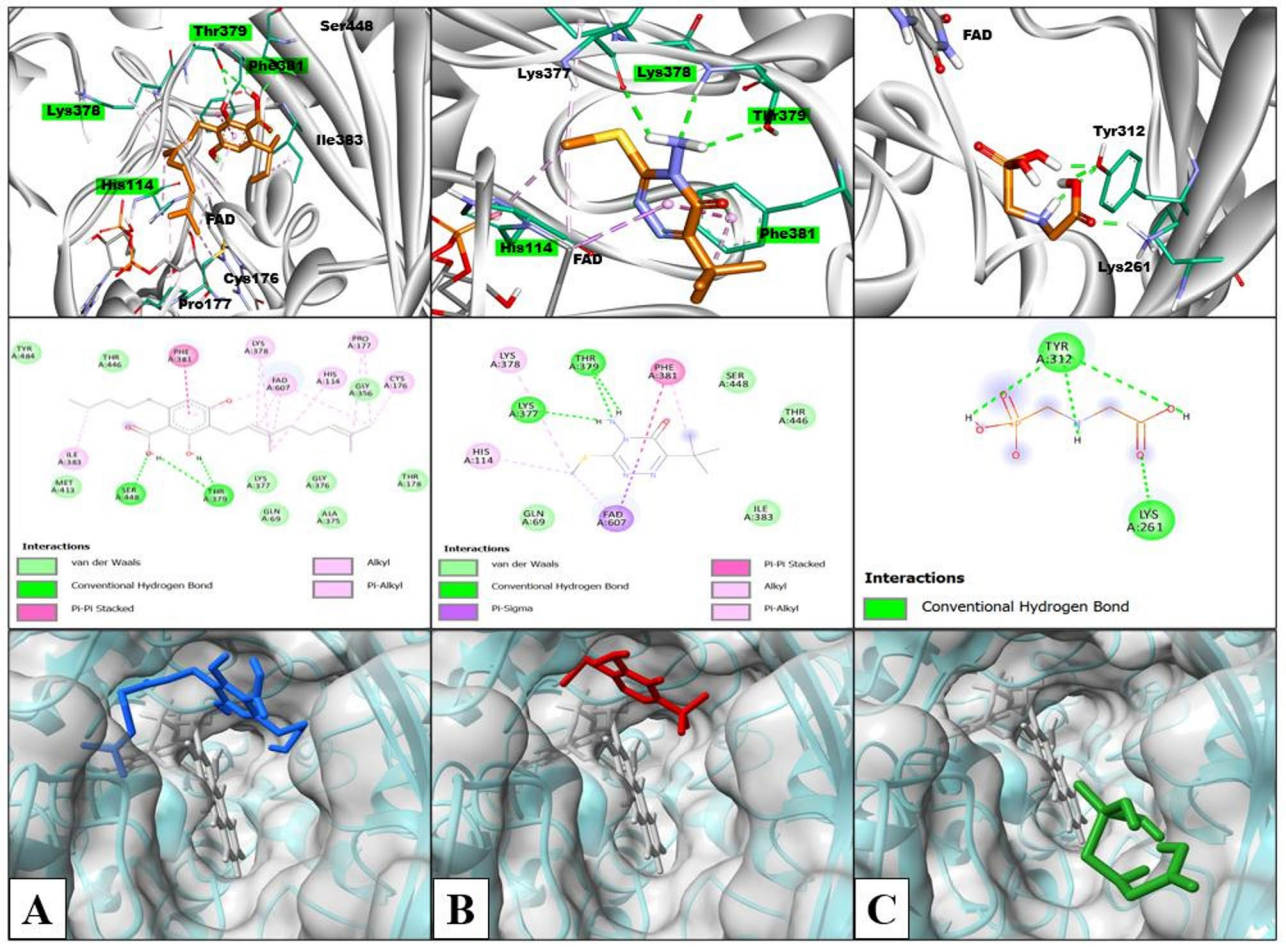
Visualization of docking results and interacting residues (**A**) CBGA, (**B**) Metribuzin, and (**C**) Glyphosate. The top row shows the 3D interactions of ligands with amino acid residues (green) and FAD (grey); Carbon atoms: rust, nitrogen atoms: light purple, oxygen atoms: red, and sulfur atoms: yellow; common amino acid residues among CBGA and metribuzin are highlighted in green, the middle row shows types of interactions, and the bottom row shows the surface view of the active site with the docked ligands

Molecular docking studies were performed to understand the impact of varying glyphosate and metribuzin herbicide concentrations on the biosynthesis of THC by analyzing their possible interactions with the active site of THCA synthase. THCA synthase is the enzyme that converts the CBGA precursor into THC, hence CBGA was also docked with THCA synthase, and the docking model is shown in (Fig. 8). (Fig. 9) shows the docking model of metribuzin and glyphosate at the same site respectively.

**Table 1:** Docking results of CBGA, metribuzin, and glyphosate with THCA synthase along with interacting amino acids

Ligand	Interacting amino acids (H-bond)	Hydrophobic interaction	van der Waal’s interaction	Number of hydrogen bonds	Binding Affinity (Kcal/mol)
CBGA	Thr 379, Ser 448	FAD, His 114, Lys 378, Phe 381, Cys 176, Pro 177, Ile 383	Gln 69, Thr 446, Thr 178, Gly 356, Ala 375, Gly 376, Lys 377, Met 413, Tyr 484,	3	-6.06
Metribuzin	Thr 379, Lys 377	FAD, His 114, Lys 378, Phe 381	Gln 69, Thr 446, Ser 448, Ile 383	3	-5.79
Glyphosate	Lys 261, Tyr 312	NA	NA	4	-2.87

Metribuzin was completely embedded in the active site and showed interactions with the same amino acids as CBGA. Both metribuzin and CBGA formed conventional hydrogen bonds, alkyl, and π-alkyl interactions with Thr 379, Lys 378, and His 114, respectively. Phe 381 interacted with CBGA and metribuzin with π − π stacked interactions and an additional π-Alkyl interaction with metribuzin. FAD, responsible for the core catalytic activity of enzyme THCA synthase, formed alkyl interaction with CBGA and metribuzin. On top of that, it also formed π-alkyl interaction with CBGA and π-π interaction with metribuzin. Moreover, both CBGA and metribuzin interacted with Thr 446, Ile 383, Ser448, Lys 377, and Gln 69 to form alkyl, conventional hydrogen bond, and van der Waal’s interactions. Therefore, metribuzin and CBGA bind at the same active site with similar kind of interactions with binding energies of -5.79Kcal/mol and  -6.06Kcal/mol respectively. From the docking result, it is clear that the interaction of metribuzin is slightly lower than the CBGA; but both are bound deep inside the active site as there are slight differences in their binding energies. However, glyphosate was observed to be present at the entrance of the active site as its binding energy is less (-2.87Kcal/mol) as compared to the former two i.e., metribizin and CBGA. Glyphosate interacted with Tyr 312 and Lys 261 amino acid residues of THCA synthase by forming conventional hydrogen bonds as shown in Table 1. The same observation was obtained from the GC-MS analysis that supports our in-silico result.

### Glyphosate vs. Metribuzin at the recommended dose

The studied parameters showed detrimental effects in terms of herbicide stress. The comparison at the recommended dose showed glyphosate had a severe impact on RWC (30.3%), photosynthetic pigments (2.95-fold reduction for total chlorophyll, 1.77-fold reduction for carotenoid), anthocyanin (1.8-fold reduction), carbohydrate (2.39-fold reduction), catalase (3.6-fold increase) and MDA (3.0-fold increase). However, an exception in protein (2.29-fold reduction), sucrose (3.9-fold reduction), and peroxidase (5.2-fold increase) were observed. THC, an important pharmaceutical compound was impacted more by metribuzin (7.9% relative peak area) than by glyphosate (9.33% relative peak area) as observed by GC-MS analysis. Thus, we can conclude that comparatively glyphosate impacts the morpho-physiological and primary metabolites more with some exceptions whereas metribuzin impacts THC production at the recommended dose.

## Discussion

The RWC indicates the extent of dehydration of leaves. Due to the treated plant’s lower water content, the overall weight shift was less pronounced even though fresh weight and water content both fell, suggesting increased cellular concentration. Another study also recorded similar results (Issaad et al. 2022), where RWC decreased with glyphosate and other herbicide applications with elevation observed at the lowest concentration.

The reduction in chlorophyll content in case of glyphosate can be attributed to an upsurge in chlorophyll degradation, as documented in the study by (Gomes et al. 2016). Similarly, the decline in carotenoids can result from the suppression of the shikimate pathway, which lowers the concentration of plastoquinone (PQ). The decrease in PQ content is significant because it functions as a cofactor for two essential enzymes in the carotenoid biosynthesis pathway: phytoene desaturase and ζ-carotene desaturase. Plastoquinone’s declining concentration therefore exerts a direct effect on carotenoid production (Gomes et al. 2017). Furthermore, blocking EPSPS inhibits chorismate, which in turn is responsible for phenylalanine synthesis and ultimately anthocyanin via the phenylpropanoid pathway. This helps to explain the decrease in anthocyanin concentration with increasing glyphosate stress (Fuchs et al. 2021). The results obtained for metribuzin treatment can be explained because Quinone A (QA) to Quinone B (QB) electron transport is impeded by metribuzin’s binding to the D1 protein on the plastoquinone binding site. Pigment production is hindered by this obstruction of electron transport, which results in electron buildup and ultimately leads to an increase in radiation. As plastoquinone remains unreduced, it causes triplet chlorophyll to build up. The triplet chlorophyll can produce singlet oxygen in the absence of efficient quenchers (Krieger-Liszkay 2004). The accumulation of singlet oxygen and triplet chlorophyll can result in lipid peroxidation, which alters the fluidity of the membrane causing oxidative damage, and consequently declining the production of chlorophyll, carotenoid, and anthocyanin (Kruk and Szymańska 2021). Similar results were observed in a study (Gomes et al. 2016), where different concentrations of glyphosate to willow trees led to a reduction of photosynthetic pigments and ultimately reduction in photosynthesis. Another study (Volova et al. 2020) showed a reduction in photosynthetic pigments while studying the biological effects of metribuzin on various weed species.

Although glyphosate does not directly affect the production of proteins in plants, it indirectly reduces protein levels by blocking the activity of the vital enzyme EPSPS. The production of phenylalanine, tyrosine, and tryptophan- essential aromatic amino acids needed for protein synthesis are curtailed as a result of this suppression (Ahsan et al. 2008). On the contrary, the metribuzin-induced decline in protein content doesn’t stem from ammonia scarcity but rather from a probable insufficiency in its conversion into organic forms. Metribuzin inhibits the enzyme system involving glutamine synthetase (GS) and glutamine-2-oxoglutarate aminotransferase (GOGAT) which are pivotal for ammonia conversion causing accumulation of unconverted ammonia and impacting protein synthesis (Nemat Alla et al. 2008). Reduction in protein content was also observed in a study (Nemat Alla et al. 2008) while studying the effect of field dose application of metribuzin on 10-day-old wheat and maize seedlings. Another study (Radwan and Fayez 2016) showed that glyphosate reduced the protein content and showed other morphological symptoms in *Arachis hypogaea* leaves.

The principal action mechanism of glyphosate is to inhibit EPSPS, a key component in the shikimate pathway, for the synthesis of aromatic amino acids. The accumulation of shikimate and its derivatives because of the disturbed shikimate pathway puts the plant under oxidative stress (Yokoyama et al. 2021). Metribuzin similarly obstructs photosynthesis by suppressing photosystem II (PSII), preventing electron transfer, and causing an excess of reactive oxygen species (ROS) to be produced in plant cells (Kostopoulou et al. 2020). Antioxidant enzymes like peroxidase and catalase are upregulated as a result of the stress that triggers the plant’s defense mechanisms. The higher amounts of reactive oxygen species (ROS) brought on by the disruption in the metabolic pathways are neutralized and detoxified by these enzymes. Nevertheless, the imbalance brought on by herbicide stress can outweigh the plant’s defense mechanisms despite the antioxidant response, which can lead to the building up of malondialdehyde (MDA), a sign of lipid peroxidation and cellular damage (Khaleghnezhad et al. 2021). Therefore, the simultaneous increase in MDA concentration indicates oxidative damage and disruption by herbicide application, while the increase in antioxidant enzymes demonstrates the plant’s attempt to fight oxidative stress (Karimmojeni et al. 2022). Similar results have been observed by (Eceiza et al. 2022b), where glyphosate-resistant and glyphosate-sensitive *Amaranthus palmeri* were analyzed to determine glyphosate-induced oxidative stress and found that an increase in antioxidant enzymes is associated with EPSPS inhibition. Another study (Karimmojeni et al. 2022) analyzed that at high metribuzin dose, *Echinacea purpurea* leaves showed an increase in MDA levels. Similarly, while studying the toxicity assessment of metribuzin on *Vigna radiata*, it was found that metribuzin-treated plants induced antioxidant enzyme activities (Kumar et al. 2023).

Distinct in their methods, glyphosate and metribuzin cause a fall in the quantities of carbohydrates and sucrose through targeted interference with basic metabolic processes. The inhibition of EPSPS by glyphosate and inhibition of photosystem II by metribuzin results in the reduction of photosynthesis, which explains the decrease of carbohydrate and sucrose levels under herbicide stress (Pline et al. 2003). Change in the photosynthetic process directly impacts the concentration of carbohydrate and sucrose levels (Vital et al. 2017). Analogous results were observed in a study (Razieh Rajabi 2012), where the foliar application of metribuzin resulted in carbohydrate reduction. Another study (Salman et al. 2016) was conducted, where the effect of glyphosate on biochemical features of *Oscillatoria limnetica* resulted in a marked decrease in carbohydrates. Various other studies (Beker Akbulut et al. 2018; Vital et al. 2017), also noticed a reduction in sugar levels in glyphosate-treated sunflower and maize plants.

The unexpected results observed at the lowest herbicide concentration can be explained through hormesis, a dose-response phenomenon wherein low doses promote biological responses while high doses lead to their inhibition. Various herbicides like glyphosate at low concentrations can induce plant growth regulation through a variety of mechanisms like auxin production, antioxidant defense, and anatomical modifications, affecting rhizosphere cation transporters (Islam et al. 2017). Lower glyphosate levels hinder the shikimic acid pathway, which lowers lignin and promotes better plant growth (Nguyen et al. 2016). In the current study, the hormesis effect due to metribuzin has been documented for the first time. Herbicide sub-doses promote physiological and enzymatic processes as well as plant growth (Brito et al. 2018). Plant growth and function are ultimately enhanced by this adaptive mechanism, which is triggered by exposure to low-stress levels and activation of cellular defense mechanisms (Jalal et al. 2021).

The bioactive compounds identified via GC-MS analysis in our study were similar to compounds observed in other studies (Tayyab and Shahwar 2015; Vadhel et al. 2023; Wishart et al. 2024). 2,4-DTBP, despite being identified in at least 169 species of organisms, there is yet no explanation as to why organisms produce 2,4-DTBP, a toxic lipophilic phenol. A recent study revealed that healthy rice plants show similar levels of 2,4-DTBP as shown by viral infection and insect herbivory samples (Zhao et al. 2020). Nevertheless, more experimentation is required to confirm the correlation between stress and 2,4-DTBP production.

Plants, being sessile organisms, strategically utilize fatty acid production as a crucial part of their adaptive stress response to minimize the negative impact (He and Ding 2020). Many aliphatic chemicals, such as membrane glycerolipids, TAG, cutin/suberin, jasmonates, and nitroalkenes (NO2-FAs), are produced by plants using fatty acids as raw material. All these compounds including fatty acids contribute to plant defense against different biotic and abiotic stressors (He and Ding 2020; de Oliveira et al. 2022). According to a study (Filimonova et al. 2018), there are reports of herbicides having ecotoxicological and biochemical effects on a variety of marine plankton species, leading to an increase in the proportion of fatty acids in response to toxicity. The mechanisms may include alteration in the *de novo* synthesis of fatty acids through fatty acid synthetase enzyme and/or via modification of existing lipids via desaturase enzyme action. In a study, an increase in ~ 17% lipid content was observed in glyphosate-treated *Chlorella sorokiniana* as compared to the control (Jaiswal et al. 2020). The effects of metribuzin on fatty acid synthesis are not well documented yet, but fatty acid synthesis is an energy-expensive process that requires ATP and NADPH at different steps; thus, it can be hypothesized that blocking of electron transport chain by metribuzin reduces energy production which in turn affects production of fatty acids. In addition, delay in electron transport from QA to QB results in over-reduction on the PSII acceptor side, which raises the chances of producing reactive oxygen species as explained in the photosynthetic pigments section, leading to lipid peroxidation in thylakoid membranes, resulting in a decrease in fatty acids (Roncel et al. 2007). This complex interaction highlights the diverse ways in which plants react to herbicidal stresses and advances our knowledge of the complex mechanisms underlying plant adaptability.

The impact of herbicides on cannabinoids can be plausibly explained by the observed relationship between herbicide treatments and fatty acid synthesis as the cannabinoids are synthesized from Geranyl pyrophosphate (GPP), a precursor derived from Acetyl-CoA via the fatty acid synthesis pathway (Macherone 2020). On the other hand, the decrease in THC at the lowest concentration of glyphosate is due to the biosynthesis of another compound CBD at the same treatment since THC and CBD are synthesized from the same precursor (CBGA); so even if the precursor were produced in greater concentrations as can be correlated from higher synthesis of fatty acids, the precursor would be utilized by the plant in the production of CBD in addition to THC. According to various studies, the synthesis of cannabinoids in plants varies under different environmental conditions. Drought stress, for example, was associated with an increase in cannabinoid production (Caplan et al. 2019). Similarly, a study reported that the active compound THC increased when the plants were exposed to UVB, whereas identical conditions did not cause any changes in the plant’s cannabinoids (Lydon et al. 1987). Copper was also found to cause a significant temporal increase in the production of THC and CBD (Cahill et al. 2024). Abscisic acid has been implicated in affecting THC concentration in plants, although its effect remains controversial (Mansouri and Asrar 2012). Contrastingly, cannabinoid levels were observed to decline under herbicide stress (Toth et al. 2021). From these observations, it can be concluded that there is a complex relationship between environmental factors and upsurge and decline in cannabinoid levels which has not yet been studied in detail and needs further investigation.

Furthermore, in-silico studies provided valuable insights into the interactions of metribuzin and glyphosate with the target- THCA synthase enzyme. The interaction between the target enzyme and the ligand is influenced by the physicochemical properties and molecular structure of the ligands. The enzymatic activity of THCA synthase occurs in a hydrophobic environment (Rodziewicz et al. 2019), which is consistent with the minimal topological polar surface area (TPSA) of its substrate CBGA. Compared to glyphosate, metribuzin possesses a lower TPSA value, suggesting metribuzin may more freely access and bind to THCA synthase as compared to glyphosate. Based on the physicochemical and optimization analysis, it was observed that metribuzin is more similar to CBGA than glyphosate. Molecular docking studies elucidated the impact of glyphosate and metribuzin on the biosynthesis of THC by analyzing their possible interactions with the active site of THCA synthase (Shoyama et al. 2012). The comparative analysis of the three docking models provides valuable insights into the mechanisms of metribuzin and glyphosate in THC production. The models demonstrate that metribuzin directly competes with CBGA for binding to the active site of THCA synthase. Hence, it might be possible that metribuzin hinders the activity of CBGA by preventing CBGA from binding at the active site of THCA synthase and consequently reduces THC biosynthesis with an increase in metribuzin stress. Moreover, at higher metribuzin concentrations, there might be a reduction in CBGA production through inhibition of the fatty acid synthesis pathway that supplies CBGA. This dual impact of metribuzin, reducing both CBGA production and binding, leads to low activity of THCA synthase and the lowest area percent of THC at the highest concentration (T4) as compared to other treatment groups. In contrast, glyphosate partially blocks the entry to the active channel of THCA synthase, hindering CBGA binding to the active site, and thus resulting in a significant reduction in THC synthesis. The increase in fatty acids from glyphosate treatment produces a slight upward trend in THC levels due to the increased availability of CBGA. From our in-silico investigation, it can be concluded that while glyphosate partially blocks CBGA entry, still causing an increase in THC production due to precursor quantity enhancement, metribuzin leads to a downward trend in THC synthesis by binding to the THCA synthase active site and decreasing CBGA substrate availability through fatty acid pathway inhibition. Thus at the highest herbicide concentration, metribuzin has more impact on the cannabis plant’s THC production as compared to glyphosate as evident from the experimental data and supported by the in-silico data analysis.

The influence of herbicides on cannabinoid biosynthesis aligns with other documented stress responses in *Cannabis sativa*. For instance, nutrient imbalances such as P and K deficiency have been linked to increased THC production, suggesting that moderate nutrient stress can act as an elicitor (Saloner and Bernstein 2022a). In contrast, salinity and high ammonium supply suppress cannabinoid levels (Baas and Wijnen 2023a; Saloner and Bernstein 2022b), indicating that not all stress types trigger beneficial responses. Such opposing responses highlight the nuanced regulation of secondary metabolism under stress. Our findings are consistent with this complex dynamic, where herbicides, as abiotic stressors, potentially function as elicitors of metabolic reprogramming. Future research should systematically explore the thresholds at which stress transitions from beneficial to detrimental in terms of cannabinoid yield and quality.

## Conclusion

Current research work investigated the impact of glyphosate and metribuzin herbicides on *Cannabis sativa* plants. We observed that both herbicides significantly affected the production of primary and secondary metabolites, including pigments and sugar content. Antioxidant activities also increased with rising herbicide doses. However, at the lowest doses, hormesis occurred with both herbicides. Additionally, glyphosate treatment boosted fatty acid synthesis in cannabis, while metribuzin had the opposite effect. Overall, our findings indicate that herbicide stress impacts overall cannabis productivity and alters biosynthesis. The stress notably stimulates the production of cannabidivarol and cannabidiol, although the reasons behind this require further investigation. In addition, molecular docking studies revealed that metribuzin binds to the same active channel as CBGA- the THC precursor, while glyphosate binds at the entrance, thereby hindering the THC production. This comparative analysis of biochemical and in silico studies elucidates key factors in the disruption of THC biosynthesis by glyphosate and metribuzin.

## Electronic supplementary material

Below is the link to the electronic supplementary material.

Additional file 1. Title of data: Supplementary Tables and Figures for Molecular Optimization and Docking. Description: This file includes additional tables and figures referenced in the Results section, such as energy minimization data, grid box values, and structural configurations for CBGA, metribuzin, and glyphosate (Supplementary Tables S1–S6 and Figures S1–S4)

## Data Availability

Data is provided within the manuscript or supplementary information files.
