# Supplementary material for: Unravelling herbicide stress and its impact on metabolite profiling in Cannabis sativa: an investigative study
Source: J Cannabis Res. 2025 Jul 7;7:40. doi: 10.1186/s42238-025-00300-z (PMC12236017; doi:10.1186/s42238-025-00300-z)
Supplement: Supplementary file 1 — Additional file 1. Title of data: Supplementary Tables and Figures for Molecular Optimization and Docking. Description: This file includes additional tables and figures referenced in the Results section, such as energy minimization data, grid box values, and structural configurations for CBGA, metribuzin, and glyphosate (Supplementary Tables S1–S6 and Figures S1–S4) [file 42238_2025_300_MOESM1_ESM.docx]

**S1 Table: Grid box values for THCA synthase enzyme.**

| **Ligands** | **Centre** | | | **Dimensions** | | |
| --- | --- | --- | --- | --- | --- | --- |
|  | **X** | **Y** | **Z** | **X** | **Y** | **Z** |
| CBGA | 40.12 | 39.992 | -27.502 | 126 | 126 | 88 |
| Metribuzin | 41.44 | 42.887 | -27.502 | 126 | 126 | 88 |
| Glyphosate | 40.29 | 43.683 | -27.502 | 126 | 126 | 88 |

**S2 Table: Physicochemical properties comparison of CBGA, metribuzin, and glyphosate.**

| Name | Chemical formula | Structure | MW (g/mol) | HBD | HBA | RB | AR | TPSA | PubChem CID Reference |
| --- | --- | --- | --- | --- | --- | --- | --- | --- | --- |
| CBGA | C_22_H_32_O_4_ | 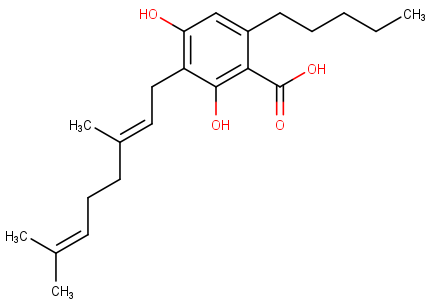 | 360.5 | 3 | 4 | 13 | 1 | 77.8 | 6449999 |
| Metribuzin | C_8_H_14_N_4_OS | 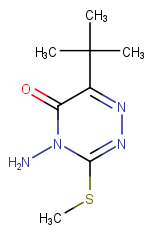 | 214.29 | 1 | 5 | 3 | 1 | 96.4 | 30479 |
| Glyphosate | C_3_H_8_NO_5_P | 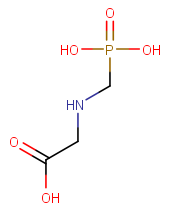 | 169.07 | 4 | 6 | 7 | 0 | 107 | 3496 |

| **Software (Force field)** | **Chem3D Pro (MM2)** | | **Chem3D**  **(MM2)** | | **Avogadro (MMFF94)** | | **Chem3D (MMFF94)** | |
| --- | --- | --- | --- | --- | --- | --- | --- | --- |
| **Energy (kcal/mol)** | **Start** | **Minimized** | **Start** | **Minimized** | **Start** | **Minimized** | **Start** | **Minimized** |
| CBGA | 1203.416 | 14.5137 | 17.446 | 14.5 | 72.0069 | 39.4885 | 39.462 | 39.462 |
| Metribuzin | 383.008 | 11.131 | 102.9129 | 15.75 | 161.3568 | 112.6683 | 39.602 | 69.732 |
| Glyphosate | 31.276 | 13.6115 | 18.4667 | 13.61 | -7.3735 | -23.961 | -23.945 | -23.9493 |

**S3 Table:** **Start and minimized energy (kcal/mol) after optimizing sequentially in the given order using different softwares. The green highlight shows the most minimized energies of the ligands.**

**S4 Table:** **Ligand parameters obtained from Gaussian optimization.**

| **Ligands** | **Start energy (kcal/mol)** | **Minimized energy (a.u.)** | **Dipole Moment (Debye)** |
| --- | --- | --- | --- |
| CBGA | 14.5137 | -1158.837799 | 2.1327 |
| Metribuzin | 11.131 | -1005.813539 | 9.2666 |
| Glyphosate | -23.961 | -891.6263467 | 2.9354 |

**S5 Table: Ground state configurations of CBGA, metribuzin, and glyphosate with every atom’s spatial arrangement: bond length and angle.**

| **CBGA** | | | | | |
| --- | --- | --- | --- | --- | --- |
| **Bond Length (Å)** | **Unoptimized** | **Optimized** | **Bond Angle (°)** | **Unoptimized** | **Optimized** |
| C-C (17) | 1.54 | 1.54 | H-C-H (19) | 109.47125 | 109.47116 |
| C=C (5) | 1.3552 | 1.3552 | C-C-H (43) | 109.47122 | 109.47113 |
| C=O | 1.2584 | 1.2584 | C-C-C (9) | 109.47122 | 109.47122 |
| C-O (3) | 1.43 | 1.43 | C=C-C (12) | 120 | 120 |
| C-H (29) | 1.07 | 1.07 | C-C-O (2) | 120 | 120 |
| O-H (3) | 0.96 | 0.96 | C-O-H (3) | 109.471 | 109.471 |
|  | | | O=C-O | 120 | 120 |
|  |  |  | C=C-H (3) | 119.99986 | 118.04065 |
|  |  |  | C=C-O (2) | 120.00007 | 113.89094 |
|  |  |  | C-C=O | 120.00008 | 128.23227 |

| **Metribuzin** | | | | | |
| --- | --- | --- | --- | --- | --- |
| **Bond Length (Å)** | **Unoptimized** | **Optimized** | **Bond Angle (°)** | **Unoptimized** | **Optimized** |
| C-C (5) | 1.54 | 1.54 | H-C-H (12) | 109.47122 | 109.47122 |
| C=N (2) | 1.30531 | 1.30531 | H-S-C (3) | 109.47122 | 109.47122 |
| C=O | 1.2584 | 1.2584 | C-C-C (7) | 109.47122 | 109.47122 |
| C-H (12) | 1.07 | 1.07 | C-C=O | 120.00009 | 120.00009 |
| N-N (2) | 1.40701 | 1.40701 | C-O-H | 109.47127 | 109.47127 |
| C-N (1) | 1.47526 | 1.37124 | C-S-C | 109.47122 | 99.02128 |
| N-C (1) | 1.46380 | 1.40647 | S-C=N | 119.04398 | 116.21160 |
| C-S (2) | 1.78000 | 1.76828 | C=N-N (2) | 123.07735 | 118.50765 |
| N-H (2) | 1.00000 | 1.01775 | C-C=N (2) | 118.92519 | 112.36916 |
|  | | | C-N-N (2) | 123.07735 | 109.38199 |
|  |  |  | C-N-C | 110.31853 | 110.31856 |
|  |  |  | N-C-S | 119.03777 | 119.03777 |
|  |  |  | O=C-N | 120.6129 | 120.6129 |
|  |  |  | N-N-H (2) | 109.47122 | 106.98, 107.67 |

| **Glyphosate** | | | | | | |
| --- | --- | --- | --- | --- | --- | --- |
| **Bond Length (Å)** | **Unoptimized** | **Optimized** | **Bond Angle (°)** | | **Unoptimized** | **Optimized** |
| P=O | 1.5048 | 1.47966 | O-P-O | | 109.4712 | 104.6909 |
| P-OH | 1.71 | 1.62665 |  |  |  | 113.3118 |
|  |  | 1.62035 |  |  |  | 114.4972 |
| P-C | 1.82 | 1.82095 | O-P-C | | 109.4712 | 120.4209 |
| C-N | 1.47 | 1.45318 |  |  |  | 99.88669 |
|  |  | 1.45504 | O=P-C | | 109.47122 | 120.42088 |
| C-H | 1.07 | 1.10725 | P-C-N | | 109.4712 | 109.93572 |
|  |  | 1.10285 | C-N-C | | 109.4712 | 113.29973 |
|  |  | 1.09412 | N-C-C | | 109.4712 | 112.89148 |
|  |  | 1.09165 | C-C-O | 120 | | 106.20121 |
| C-C | 1.54 | 1.51562 | C-C=O | 120 | | 124.92402 |
| C=O | 1.2584 | 1.20155 | O=C-O | | 120.0 | 123.07 |
| C-OH | 1.43 | 1.35432 |  | | | |
| O-H (3) | 0.96000 | 0.96476 |  |  |  |  |

**S6 Table: IUPAC, chemical formula, structure, and similarity index of identified *Cannabis sativa* L. compounds.**

| Sr.No. | Identified compound | IUPAC | Chemical Formula | Chemical Structure | Similarity Index |
| --- | --- | --- | --- | --- | --- |
| 1. | Methyl palmitate | Methyl hexadecanoate | C_17_H_34_O_2_ | 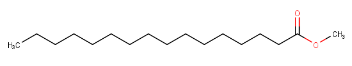 | 95% |
| 2. | Methyl stearate | Methyl octadecanoate | C_19_H_38_O_2_ | 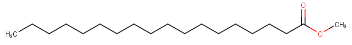 | 95% |
| 3. | 2,4-DTBP | 2,4-Di-tert-butylphenol | C_14_H_22_O | 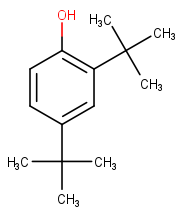 | 93% |
| 4. | Eicosane | Icosane | C_20_H_42_ | 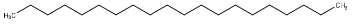 | 91% |
| 5. | Heptadecane | Heptadecane | C_17_H_36_ | 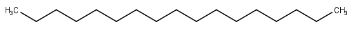 | 90% |
| 6. | Cetane | Hexadecane | C_16_H_34_ | 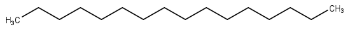 | 90% |
| 7. | delta.9-Tetrahydrocannabivarin | (6aR,10aR)-6,6,9-trimethyl-3-propyl-6a,7,8,10a-tetrahydrobenzo[c]chromen-1-ol | C_19_H_26_O_2_ | 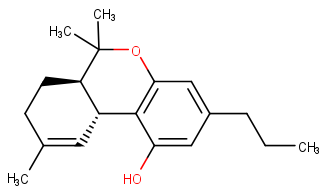 | 92% |
| 8. | Dronabinol (THC) | (6aR,10aR)-6,6,9-trimethyl-3-pentyl-6a,7,8,10a-tetrahydrobenzo[c]chromen-1-ol | C_21_H_30_O_2_ | 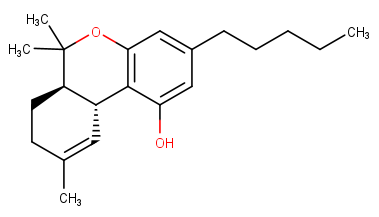 | 91% |
| 9. | Cannabinol | 6,6,9-trimethyl-3-pentylbenzo[c]chromen-1-ol | C_21_H_26_O_2_ | 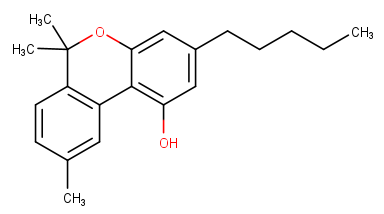 | 94% |
| 10. | Cannabidivarol | 2-[(1R,6R)-3-methyl-6-prop-1-en-2-ylcyclohex-2-en-1-yl]-5-propylbenzene-1,3-diol | C_19_H_26_O_2_ | 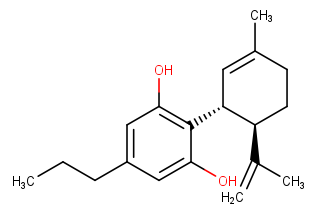 | 92% |
| 11. | Cannabidiol | 2-[(1R,6R)-3-methyl-6-prop-1-en-2-ylcyclohex-2-en-1-yl]-5-pentylbenzene-1,3-diol | C_21_H_30_O_2_ | 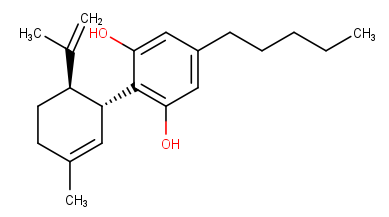 | 90% |
| 12. | Heneicosane | Henicosane | C_21_H_44_ | 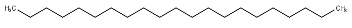 | 90% |
| 13. | Docosane | Docosane | C_22_H_46_ | 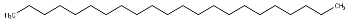 | 90% |
| 14. | Tetracosane | Tetracosane | C_24_H_50_ | 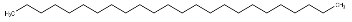 | 91% |


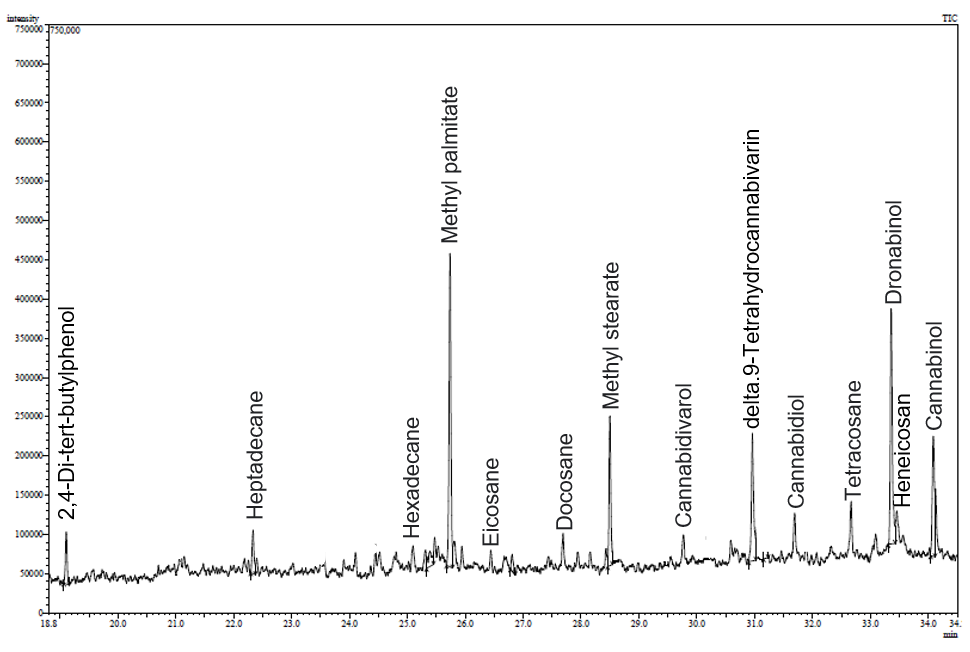


**S1 Figure:** Secondary metabolites identification in *Cannabis sativa* L. extract through GC-MS technique.


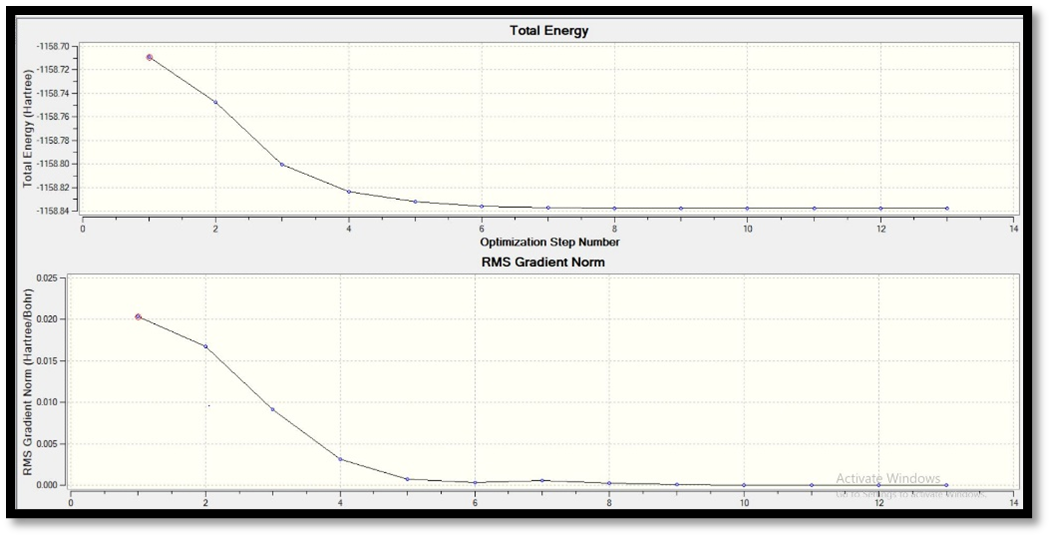


**S2 Figure:** **Showing the energy minimization and RMS gradient normalization for CBGA with optimization stem number.**


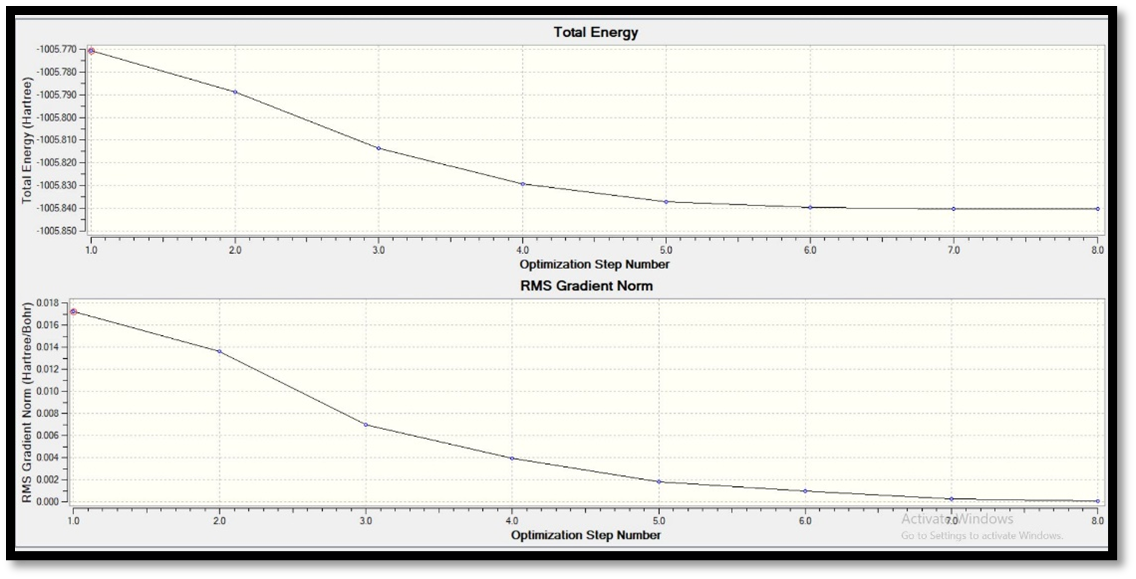


**S3 Figure:** **Showing the energy minimization and RMS gradient normalization for metribuzin with optimization stem numbers.**


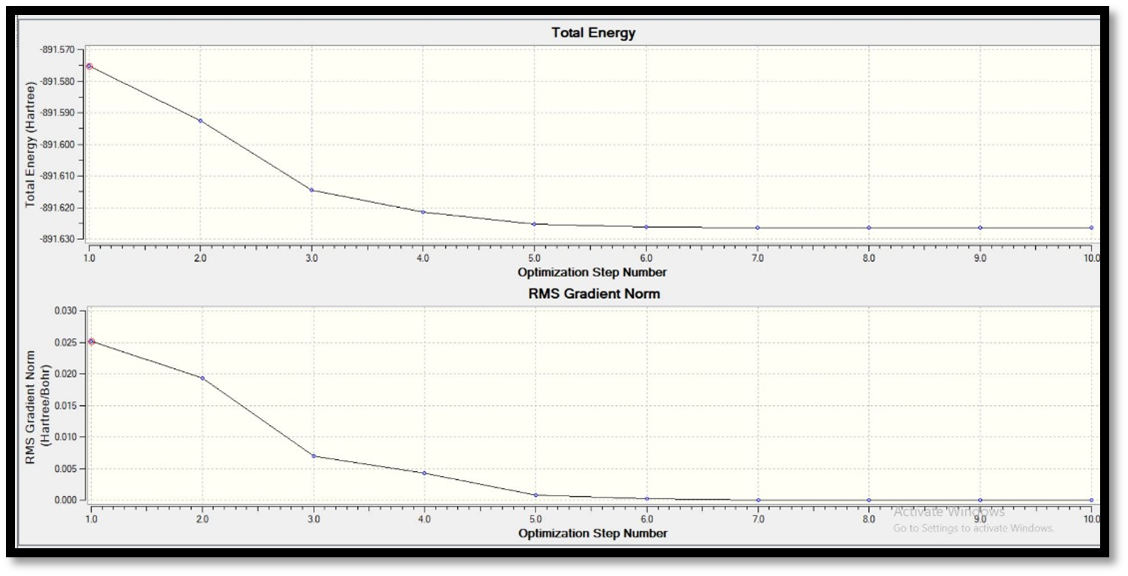


**S4 Figure:** **Showing the energy minimization and RMS gradient normalization for glyphosate with optimization stem numbers.**
